# Designing for trustworthiness and research reciprocity: An example from the Illinois research network hub in the NIH RECOVER adult cohort study

**DOI:** 10.1017/cts.2025.10050

**Published:** 2025-05-23

**Authors:** Elijah Kindred, Alejandra L. Ibañez, Marta Cerda, Emily Kowey, Hugh Musick, Robin Mermelstein, Lynn B. Gerald, Jerry A. Krishnan

**Affiliations:** 1 Bright Star Community Outreach, Chicago, USA; 2 Illinois Unidos, Chicago, USA; 3 ASI Home Care Services, Chicago, USA; 4 Office of Population Health Sciences, University of Illinois Chicago, Chicago, USA; 5 Breathe Chicago Center, Division of Pulmonary, Critical Care, Sleep, and Allergy, University of Illinois Chicago, Chicago, USA; 6 Institute for Health Research and Policy and Psychology Department, University of Illinois Chicago, Chicago, USA

**Keywords:** Covid-19, community-based organization, engagement, recruitment, retention

Community-based participatory research (CBPR) is a collaborative model of translational research [1] that engages community members and researchers as equal partners, focused on addressing community-identified needs to drive policy change.[2–4] Increasingly, early-stage translational researchers partner with affected populations, including community-based organizations (CBOs), to identify and overcome barriers to recruitment and retention [5,6]. While such a role is critical, limiting CBOs involvement to recruitment and retention views these organizations’ primary function as serving as an intermediary between researchers and participants and fails to appreciate the full scope of what partner organizations can offer to research collaborations.

We advocate for expanded inclusion of CBOs in designing early-stage translational research as a part of a paradigm shift to designing for dissemination and sustainability (D4DS) [7]. D4DS refers to principles and methods for ensuring the fit between a health innovation and the context before adoption. A co-design process including researchers and CBOs can ensure that innovations are usable by the communities which are the target of the intervention [7–11]. This report describes a unique example of a community-led summit which brought together patients, community organizations, federal and state organizations, and state legislators to address community concerns regarding Long COVID. To our knowledge, this is the first example of community involvement on a national scale for a study that spans multiple methodologies including pathogenesis, epidemiology, and clinical trials. The NIH Researching COVID to Enhance Recover (RECOVER) Initiative aims to deepen our understanding of Long COVID’s epidemiology and develop effective treatments for affected individuals [12]. RECOVER is one of the largest studies ever funded by the federal government representing $1.7 billion in investment since its inception in 2021. Here, we highlight how CBOs leveraged their access to scientific experts and healthcare providers to elevate awareness of affected communities’ needs, organizing a national meeting with local, state, and federal policymakers.

The Illinois Research Network (ILLInet) Hub, a collaborator in the RECOVER Adult Cohort Study, operates across five Illinois sites, including two operated by CBOs (Illinois Unidos and Bright Star Community Outreach) [13]. ILLInet includes seven additional CBOs (ASI Home Care Services, Envision Community Service, Teamwork Englewood, Tri-County Urban League, Peoria Friendship House, Chicago Urban League, Central Illinois Friends) who contribute by co-designing and disseminating educational materials on COVID-19 and Long COVID, providing feedback on community-based research activities, and identifying effective communication channels for outreach. These partnerships were essential to ILLInet’s enrolling and conducting follow-up visits in 900 diverse participants (49% non-Hispanic white, 22% non-Hispanic Black, 14% Hispanic, 6% multiple, 5% Asian, 3% unknown).

In the study’s fourth year, three CBOs (ASI Home Care Service, Bright Star Community Outreach, and Illinois Unidos) organized a summit to learn about research findings and engage state policymakers. The summit featured 61 in-person and 367 virtual attendees (Table 1) and included live Spanish interpretation. People with Long COVID, representatives of community organizations, and federal and state government officials served as speakers and discussants on various topics, including the 2024 National Academies’ definition of Long COVID [14], the NIH RECOVER Initiative (observational studies and clinical trials) [12], strategies for stakeholder engagement and policy advocacy[15], barriers to Long COVID care[16], and the AHRQ Long COVID Care Network [17].

**Table 1.** Stakeholder groups participating in the long COVID summit.^1^

	In Person (n = 61)	Virtual (n = 374)
**COMMUNITY-BASED ADVOCACY AND SERVICE ORGANIZATIONS**		
Access Living		X
Arab American Family Services		X
ARISE Chicago		X
Black COVID Survivors Group[2] (Teresa Akintowa)	X	X
Blancarte Strategies		X
Bright Star Community Outreach[2] (Elijah Kindred)	X	X
Carole Robertson Center for Learning		X
Centro San Bonifacio		X
Chicago Foundation for Women	X	
Chicago Jobs with Justice		X
Colorado Long COVID Coalition		X
CORe Community, Inc. (COVID Recovery through Community)		X
COVID-19 Longhauler Advocacy Project		X
COVID Recovery Through Community		X
Despertar Latino		X
Dupage Federation on Human Services Reform		X
ELLAS		X
Enlace Chicago		X
Ever Thrive Illinois	X	
Generations Health Care Initiatives		X
Greater West Town Community Development Project		X
Healthy Communities Foundation	X	
Hispanic Federation		X
Illinois Partners for Human Service		X
Illinois Unidos[2] (Alejandra Ibanez)	X	X
Latino Alzheimer’s and Memory Disorders Alliance		X
Massachusetts ME/CFS & Fm & Long COVID Support Association		X
Massachusetts League of Community Health Centers[2] (Dr Cheryl Clark)	X	X
ME Action		X
Medical Organization for Latino Advancement	X	
Medicine Song Woman Creations		X
Minnesota ME/CFS Alliance		X
National Coalition for Latinx with Disabilities	X	
National Latino Coalition Against COVID[2] (Dr Daniel Turner-Lloveras)	X	
National Organization of Nurses with Disabilities		X
Native Interwoven Tribal Community		X
Padres Angeles	X	
Patient Advocate Foundation		X
Project HOOD		X
Proyecto de Acción de los Suburbios del Oeste West Suburban Action Project	X	
Puerto Rican Cultural Center		X
Respiratory Health Association	X	X
The Resurrection Project		X
Safe Works Access Program		X
Safer Foundation		X
SER/SERCO Central States		X
SGA Youth and Family Services		X
Solve ME		X
Southeast Chicago Commission		X
Southern Illinois Independent Living[2] (Beth Finta)	X	
Southwest Organizing Project		X
Youth Guidance		X
**PATIENTS WITH LONG COVID & RECOVER STUDY PARTICIPANTS**	X	X
**FAITH GROUPS**		
Church of Jesus Christ		X
**HEALTH CARE PROVIDERS**		
***Clinicians***		
The Adaptive Behavior Institute		X
Dr Byrd Family Practice		X
Goodenberger Counseling Solutions		X
***Federally Qualified Health Care Centers***		
Alivio Medical Center		X
Erie Family Health Centers		X
Pillars Community Health		X
***Home Care and Rehabilitation Services***		
ASI Homecare[2] (Planning Committee) Marta Cerda	X	X
Dorothy’s Care		X
Habilitative Services, Inc		X
Illinois Department of Public Health, Division of Rehab Services[2] (Rahnee Patrick)	X	
Southern Illinois Center for Independent Living[2] (Beth Finta)	X	
Vermont Center for Independent Living		X
***Hospitals and Health Systems***		
Advocate Christ Medical Center		X
Advocate Health		X
Children’s National Hospital		X
Jesse Brown VA		X
Loretto Hospital		X
Mount Sinai West/Morningside		X
New York University, Langone		X
OSF Healthcare Saint Francis Medical Center		X
University of Illinois Health[2] (Dr Robert A. Barish; Lauren Kraus)	X	X
University of Illinois Chicago Office of Community Engagement and Neighborhood Health Partnerships		X
University of North Carolina Long COVID Recovery Clinic		X
VA Palo Alto Health Care		X
***Payors***		
Illinois Medicaid[2] (Dr Arvind Goyle)	X	
***Other***		
AcuPlus Wellness Center		X
Lawrence Hall		X
Neuroversion, Inc		X
**INDUSTRY**		
Timeless Biosciences		X
**MEDIA**		
The New York Times		X
**POLICY-MAKERS AND QUALITY IMPROVEMENT ORGANIZATIONS**		
Chicago Community Health Response Corps		X
Chicago Department of Public Health[2] (Drs. Olusimbo Ige and Geraldine Luna)	X	X
Colorado Department of Public Health and Environment		X
Consulado General de México en Chicago		X
Illinois Department of Healthcare and Family Services	X	
Illinois Department of Human Services[2] (Dr Arvid Goyal)	X	
Illinois Department of Public Health[2] (Dr Catherine Counard)	X	
Illinois Department of Rehabilitative Services	X	
New York City Department of Health and Mental Hygiene		X
Office of Illinois State Senator Julie Morrison – 29th Legislative District		X
Illinois State Senator Karina Villa[2] – 25th Legislative District (Karina Villa)		
Office of Illinois State Senator Mattie Hunter – 3rd Legislative District[2] (Mattie Hunter)	X	
Office of Long COVID Research and Practice, Department of Health and Human Services[2] (Allison O’Donnell)	X	
TMF Health Quality Institute		X
US Department of Health and Human Services		X
**PROFESSIONAL ASSOCIATIONS**		
American Board of Internal Medicine		X
American Heart Association		X
Association of the Scientific Medical Societies in Germany		X
**RESEARCH ORGANIZATIONS**		
Agency for Healthcare Research and Quality[2] (Dr Poonam Pardasaney)	X	
Arizona State University		X
Boston COVID Recovery Cohort[2] (Dr Cheryl Clark)		X
Breathe Chicago Center		X
Brigham and Women’s Hospital		X
Carnegie Mellon University		X
Dartmouth Hitchcock Medical Center		X
Duke University, School of Medicine[2] (Dr Kanecia Zimmerman)	X	
Emory University, School of Medicine[2] (Dr Zanthia Wiley)	X	
Howard University		X
Johns Hopkins University, School of Medicine[2] (Dr Alba Azola)	X	
Loyola University		X
National Institutes for Health		X
Next Innovative Clinical Research		X
New York University, Grossman School of Medicine		X
Northwestern University, Feinberg School of Medicine	X	
Nova Southeastern University		X
RECOVER National Community Engagement Group[2] (Felicia Blakley, Marta Cerda, Alejandra Ibinez)	X	
Rosalind Franklin University of Medicine and Science	X	
Stanford University, College of Medicine[2] (Dr Jonathan Klein)	X	
Tanoma Consulting	X	
University at Buffalo Jacobs School of Medicine and Biomedical Sciences		X
University of California, Davis		X
University of California, San Francisco[2] (Dr Neeta Thakur)	X	
University of Cincinnati		X
University of Colorado Anschutz Medical Center		X
University of Connecticut, School of Medicine[2] (Dr Linda Sprague Martinez)	X	
University of Illinois[2] (Theresa Eagleson, Dr Lynn Gerald, Emily Kowey, Dr Jerry Krishnan, Hugh Musick)	X	X
University of Massachusetts Medical School		X
University of Michigan		X
University of New Hampshire		X
University of North Carolina Chapel Hill		X
University of Washington		X
Vanderbilt University Medical Center		X
Washington University in St Louis		X
**ADDITIONAL STAKEHOLDERS**		
Gentle Giants Care for Homes		X
New Horizons		X
Rosenberg Strategies		X
SBC Global Consulting		X
Suleiman and Associates	X	

1 Organizations that registered for the Summit are grouped into categories to show the breadth and diversity of participants, but organizations may fit into more than one category. [2] Indicates speaker, panelist, or moderator at the Summit.

After the didactic portions of the meeting, in-person summit participants were divided into work groups for in-depth discussion to determine recommendations for actions to improve Long COVID care. The following actions were recommended:

1. *Engage* CBOs or other trusted representatives of affected populations as research partners from the start of studies involving human volunteers to ensure study activities are culturally sensitive, linguistically accessible, and address systemic barriers (e.g., mistrust in medical institutions among people of color).
2. *Fund* CBOs or other trusted representatives to co-design outreach and support (e.g., education, mask distribution), provide navigation services (e.g., disability applications, provider referrals), and share information about research opportunities, including clinical trials.
3. *Expand* the AHRQ Long COVID Care Network and increase funding for training healthcare professionals to effectively diagnose, treat, and advocate for Long COVID patients.
4. *Support* researcher-CBO collaborations to drive policy change and combat misinformation, enhancing trust and promoting health equity.

CBO partners are presenting these recommendations to the Illinois State Legislature which are more likely to be adopted because CBOs and researchers worked together. The recommendations are based on science but also consider the needs and desires of the communities most affected by Long COVID. CBOs have substantial influence in lobbying legislators and public health officials to adopt and sustain programs and policies.

We conclude that the current model of CBPR should be expanded to support greater reciprocity between early-stage translational research and affected populations. By collaborating closely with CBOs and other representatives, researchers can go beyond traditional recruitment and retention goals to conduct studies that address knowledge gaps and are designed to promote trustworthiness in both the research process and study results. CBOs can be valuable collaborators in co-designing research questions and implementing studies. These partnerships enable researchers to gain valuable insight into community needs, ensure culturally and contextually appropriate study designs, provide mechanisms for communities to engage meaningfully in research, and enhance adoption potential. Moving toward a D4DS approach enhances the public health impact of research by ensuring effective dissemination such as policy changes that benefit all populations.
